# Indeterminacy and the principle of need

**DOI:** 10.1007/s11017-016-9393-5

**Published:** 2016-12-31

**Authors:** Anders Herlitz

**Affiliations:** 1 Department of Philosophy, Linguistics and Theory of Science, University of Gothenburg, Box 100, 405 30 Gothenburg, Sweden; 2 Department of Philosophy, Rutgers University, New Brunswick, NJ USA

**Keywords:** Principle of need, Priority setting, Health care rationing, Indeterminacy

## Abstract

The principle of need—the idea that resources should be allocated according to need—is often invoked in priority setting in the health care sector. In this article, I argue that a reasonable principle of need must be indeterminate, and examine three different ways that this can be dealt with: appendicizing the principle with further principles, imposing determinacy, or empowering decision makers. I argue that need must be conceptualized as a composite property composed of at least two factors: health shortfall and capacity to benefit. When one examines how the different factors relate to each other, one discovers that this is sometimes indeterminate. I illustrate this indeterminacy in this article by applying the small improvement argument. If the relation between the factors are always determinate, the comparative relation changes by a small adjustment. Yet, if two needs are dissimilar but of seemingly equal magnitude, the comparative relation does not change by a small adjustment of one of the factors. I then outline arguments in favor of each of the three strategies for dealing with indeterminacy, but also point out that all strategies have significant shortcomings. More research is needed concerning how to deal with this indeterminacy, and the most promising path seems to be to scrutinize the position of the principle of need among a plurality of relevant principles for priority setting in the health care sector.

## Introduction

The principle of need is often proposed as a decision guide for priority setting in the health care sector. The principle is defended by Rawlsians such as Norman Daniels as well as by communitarians such as Michael Walzer, and proponents of the capabilities approach often recognize that need should guide prioritization decisions [1–3]. Regulations and guidelines in many countries rely on principles of need that might or might not be articulated [4–6]. There is also a growing interest in exploring the possibilities to spell out a useable version of a principle of need for priority setting, and some recent work has addressed different ways to aggregate need satisfaction [7–11]. A principle of need has at least two purposes in the context of priority setting. It can be applied to identify who has a claim on health resources and to establish who ought to be prioritized [1, 7]. This article contributes to the debate on the nature and usability of a principle of need for priority setting by pointing toward some innate complications with reasonable principles of need that can be used to establish prioritization issues and toward the different ways in which these can be dealt with.

The topic of the article is primarily complications around the *structure* of a reasonable principle of need when applied to priority setting situations. In order to focus on the structure of a reasonable principle of need, I will follow much philosophical work on needs and assume that a principle of need, similar to certain sufficientarian theories, promotes gap closure (i.e., health need satisfaction) and assigns relatively more weight to gap closures the worse off the beneficiary is, the more severe the health need is [12–14]. In other words, I will assume that a principle of need promotes maximization of severity-weighted need satisfaction [10]. I take this sufficientarian approach to the principle of need (a health need occurs only when there is ill-health that can be mitigated) with weighting schemes that resemble prioritarianism (the worse health someone has, the more weighty the need is) to be common and also plausible in relation to priority setting [10, 12]. Yet, some might want to conceptualize the principle of need in other ways and defend it with reference to some other normative criteria (e.g., equality, welfare maximization, health maximization). The argument presented below is, I believe, easily extrapolated to any approach to the principle of need that recognizes the importance of more than one factor, e.g., severity of need *and* need satisfaction, number of needs satisfied, *and* depth/size of the satisfied needs. The argument ought thus to be of interest to anyone interested in a principle of need (regardless of whether one believes need satisfaction is valuable in itself or valuable because it contributes to some other goal). Although, the argument might have to be modified depending on how the principle is defended, and the plausibility of different solutions to the problem that I outline below will likely depend on ones views of the role of a principle of need in a wider normative framework.

I make no argument *that* one ought to care about needs when one allocates scarce resources. Rather, I will focus on what a reasonable principle of need must be like if we care about needs, and what this means for the application of such a principle. This means that I will not say anything directly about the amount of resources that an individual who has a need is entitled to, and I will not in any length address the complex issue of when a need exists and when it matters. I agree with Niklas Juth that a reasonable principle of need must be sufficientarian in the sense that need entails an entitlement *only* to resources that will remedy ailments up to a certain level [7].

Furthermore, I believe that even if one accepts that needs are highly important for priority setting, one should accept that it cannot be the only normative consideration that applies. Equality and health maximization will, for example, enter many reasonable sets of substantive norms apt to guide prioritization decisions in the health care sector. Pointing this out already at the outset is important because it allows us to address the principle of need as a prima facie principle. The principle prescribes what one should do, but the possibility that other concerns justify an all-things-considered evaluation that is contrary to what the principle of need prescribes is very real.

Below, I will argue that a reasonable principle of need is *indeterminate* due to the complex nature of need. In the first section, I argue that in order to formulate a reasonable principle of need, one must have a conception of need, and that such a conception must take numerous different factors into account. When these factors are put together, I will argue in the second section, it is revealed that they relate to each other in a way that allows for indeterminacy. This indeterminacy spreads to the principle of need itself. In the third section of the article, I discuss what can be done in order to deal with this.

Within discussions on priority setting, a distinction between micro- and macro-levels is sometimes proposed [15, 16]. A principle of need can be applied in both micro- and macro-levels, although it might take somewhat different shapes in different spheres. The argument in this article is relevant to both macro- and micro-levels.

## Need as a composite property

Roughly stated, a principle of need prescribes that one satisfy needs and prioritize individuals with the highest, or the weightiest, most severe, needs. In order to understand and use this principle, one must know what need is and how to establish its degree, or weight, of severity.

In this article, I am mainly interested in the structure of a reasonable principle of need. Therefore, I will sidestep the difficulties surrounding the task of identifying the exact nature of what it is we have needs *for* and whether or not needs are only instrumental or can be also categorical [7, 17]. One way of thinking about the issue without having a substantive concept of what individuals need, and which I will adopt, is to introduce a numerical placeholder for that which there is a need, 1, and then to calculate degrees of need using this placeholder [10, 11, 18, 19]. This method is similar to how health economists sometimes use the quality of health concept in theorizing about QALY. A need, in other words, can be seen as a shortfall from that which matters. The exact conditions that must obtain in order for there to be no need is an issue that must be addressed, but one can theorize around the *structure* of a reasonable principle of need before answering that question.

It is clear that need is a complex notion. In the context of priority setting in the health care sector, there are at least five different types of interpretations of need: (1) need as immediate threat to life; (2) need as immediate shortfall from that which should be, e.g., good health, a set of capabilities, a certain welfare level; (3) need as lifetime shortfall from that which should be; (4) need as immediate capacity to benefit; and (5) need as lifetime capacity to benefit [20]. A person who risks losing her life due to health problems has a health need due to this risk. A person who in a specific moment has reduced health has a need due to this shortfall. A person with a prospect of reduced health in her lifetime has a need due to this shortfall. A person who in a specific moment can enjoy a health benefit has a corresponding need. And a person who can enjoy a lifetime health benefit has a corresponding need. Furthermore, one can view need satisfaction as instrumentally valuable or as a final good [17].

A possible (theoretical) solution would now be to argue in favor of one of these interpretations and suggest that one of these five is what need *really* is, and then defend either an instrumental or categorical interpretation of needs. I will not pursue that route here, as I believe it is a dead end (a reasonable concept of health need used in order to settle priority setting issues cannot be based on *either* health shortfalls or capacity to benefit) [13]. Instead, I will suggest that if one wants a single, reasonable principle of need one must accept that such a principle has to rely on a concept of need that is complex and incorporates the underlying intuitions and ethical considerations that generate the various interpretations of health need outlined above. An acceptable principle of health need must incorporate both health shortfalls and capacity to benefit from an intervention [14].

In a recent article, Tony Hope, Lars Peter Østerdal, and Andreas Hasman have underlined some difficulties surrounding principles of need that relate to how to systematically take into account the fact that certain interventions only have a certain probability to increase health, that health benefits sometimes occur long after the actual intervention, and that the best outcome for patients often is obtained through a combination of treatments [9]. These difficulties also point toward shortcomings of simpler need concepts, to how a more complex concept of need that incorporates probabilities and time lags must be developed, and to a conception of need satisfaction that has to take into account that the value of partial need satisfaction does not necessarily correspond to the proportion of the need satisfaction.

Two factors that seem to capture many of the strong intuitions we have about health needs stand out. Health needs exists when there are: (1) health shortfalls, i.e., ill-health (these might have different temporal locations and different temporal extensions), and (2) capacities to benefit (immediate and lifetime capacities to benefit are both part of this) [13]. These factors can be interpreted so that they also account for the complexities identified by Hope, Østerdal, and Hasman. Capacity to benefit can be understood in probabilistic terms. Health shortfalls can be understood as capturing the contexts, and combination, of illnesses. And with a broad concept of capacity to benefit that incorporates both lifetime and immediate capacities to benefit, as well as by accepting that lifetime shortfalls are parts of what compose a health shortfall, one can account for the fact that certain treatments generate health benefits long after they have been implemented.

A reasonable suggestion is, thus, to conceptualize health need for the purpose of priority setting and for the purpose of developing a reasonable principle of need that can be applied in prioritization decisions as a composite property made up of two factors:

$${\text{Health}}\;{\text{need}} = f\left( {{\text{health}}\;{\text{shortfall}},\;{\text{capacity}}\;{\text{to}}\;{\text{benefit}}} \right)$$

It could be suggested that both these factors are captured by the capacity to benefit. However, it is important to separate a health shortfall from the capacity to benefit since doing so reveals that the degree of a health shortfall contributes to the overall need in a different way compared to capacity to benefit. A severely ill individual can have the same capacity to benefit as a relatively healthy individual (due, for example, to technology constraints). A principle of need should be able to say that of these two, the severely ill individual has a greater health need. A reasonable prima facie principle of need would state that, prima facie, we ought to prioritize the person with the highest need = *f*(health shortfall, capacity to benefit). Applying this principle (and putting a cap on what is counted as a benefit, e.g., perfect health) would be to maximize severity-weighted need satisfaction since health shortfall captures severity of ill-health and capacity to benefit captures non-weighted need satisfaction. Yet, once it is accepted that a reasonable principle of need must take this shape, the question of how the factors relate to each other immediately appears. It is this issue that I will examine further in the next section.

## Indeterminacy

In order to develop a useable health need concept composed of health shortfall and capacity to benefit, one must somehow put these factors together. It is quite conceivable that one individual might have a large health shortfall but a relatively small capacity to benefit. How should the health needs of someone who, after a traffic accident, risks being partially paralyzed and unable to walk for the rest of her life but who might fully recover if quickly treated be ranked against the health needs of someone with schizophrenia who will never be able to hold a job, create the sort of relations with other humans that most people take for granted, and lead what is considered to be a life with normal functioning? The latter’s health shortfall is arguably larger, while the former’s capacity to benefit might be greater. Answers to this question rely on how these factors are put together.

I will, in this section, argue that once the issue of how health need factors relate to one another is examined, it must be concluded that the relation between these factors is *partially indeterminate*. In the previous section, I suggested that a reasonable principle of need ought to be composed of at least two factors. Indeterminacy arises because of the possible indeterminacy of the relation between these factors from the perspective of the so-called trichotomy thesis. Sometimes, it is not true that one individual is more, less, or equally needy as another individual. The relation between the factors would be wholly indeterminate if it were *always* the case that the relation between two alternatives could not be established according to the trichotomy <more need>, <less need>, <equal need>. It is partially indeterminate if this is *sometimes* the case [21].

Much research in value theory has recently pointed to how it is unjustified to assume the veracity of the trichotomy thesis when analyzing value relations. The trichotomy <more than>, <less than>, <equal to> does not always exhaust the set of possible relations that a factor instance can bear to another factor instance for comparative purposes. Ruth Chang has in this field defended the possibility of “parity,” a fourth value relation that like <equal to> is symmetric (if A is on a par with B, then B is on a par with A), but contrary to <equal to>, is irreflexive (A is not on a par with itself) and non-transitive (if A is on a par with B, and B is on a par with C, then it is not necessarily true that A is on a par with C). This entails that, in case A and B are on a par, it is possible that a small improvement of one alternative does not change the positive relation between the alternatives, which must follow if the alternatives are equal [22, 23]. Wlodek Rabinowicz has shown that if value is to be understood in terms of which attitude it is fitting to have, there are in fact 15 types of possible positive relations between merely two items [24]. Furthermore, it has been recognized that certain standards with which alternatives are evaluated might be semantically vague so that it is indeterminate how different alternatives meet them, similarly to how it might be indeterminate whether Danny who has thick hair that covers a small part of his head is more bald than Eric who has thin hair that covers a relatively larger part of his head [25].

How should health shortfall (henceforth, HS) and capacity to benefit (henceforth, CB) be put together? For the sake of argument, similarly to how health economists sometimes invoke the quality of health concept when calculating QALY, imagine that the degree of HS can be determined such that:

$${\text{HS}} = \left( {\left( {120-{\text{actual}}\;{\text{age}}} \right)-{\text{lifetime}}\;{\text{quality}}\;{\text{of}}\;{\text{health}}\left( {{\text{expressed}}\;{\text{on}}\;{\text{a}}\;{\text{scale}}\;1 - 0 \quad {\text{where}}\;1\;{\text{is}}\;{\text{perfect}}\;{\text{health}}} \right) \times {\text{expected}}\;{\text{life}}\;{\text{years}}} \right)$$

HS expresses how far away an individual is from a life at perfect health that endures to the age 120. The age 120 is somewhat arbitrary, but if a measure is to also take into account health problems that shorten a life, some benchmark is needed, and in our day and age 120 seems to be the boundary for human life. It is worth pointing out that this benchmark, 120 years, can be adjusted without any impact on the argument below.

Imagine, further, that CB can be determined in a QALY-inspired way so that:

- CB = ((expected lifetime quality of health after intervention × expected life years after intervention) − expected QALY generated by the individual without intervention)
- CB, thus, expresses the gains in terms of QALY that can be generated by an intervention.

Once these metrics are in place, it could be suggested that a reasonable principle of need should rely on some aggregation of them, for example, the sum of the two, HS + CB. Such an aggregative move does not have to sum up HS and CB of course. Perhaps it is reasonable to give more weight to HS than to CB. If that were the case, one could put them together by multiplying HS with 2, i.e., 2HS + CB. Similarly, one could do the opposite and give greater weight to CB by adding a multiplicator to that factor. Furthermore, one could explore various ways in which to give increasing weight to health shortfalls and/or capacity to benefit the further away a person is from perfect health status. And, if appropriate, one could introduce thresholds where certain weights are attached. In principle, there is no end to how complex the aggregation of the two factors can be made.

Yet, regardless of how complex one chooses to make the aggregation, if the relation between the factors were wholly determinate, it would be true that the aggregated HS and CB for every individual will be determinately higher, lower, or equal to the aggregated HS and CB for every other person. On scrutiny, this seems false.

One might consider a (fictional) case of two individuals, Anna and Ben, with different health needs: Anna was in a car accident and contracted a complicated spinal injury, and she has a significant health shortfall as a result. If she does not receive medical treatment, she will be paralyzed from the waist down for the rest of her life. She will be able to continue working, to have a meaningful and rewarding life, but she will have to live with the disability and all that that entails; she will be unable to walk, need assistance with quotidian activities, and so on. If she does receive treatment, she will be fully cured. Her capacity to benefit is high. Ben, on the other hand, suffers from a severe type of paranoid schizophrenia, which involves hallucinations and delusions, movement disorders, reduced feelings of pleasure, feelings of extreme suspiciousness toward others, trouble focusing, and problems with the “working memory.” This significantly reduces the quality of his health. He is unable to work, it is very hard for him to form meaningful relationships with other human beings, and he is often experiencing periods of depression because of the illness. His health shortfall is very large. Although schizophrenia is a chronic disease that cannot be fully treated, Ben could benefit, and his health be slightly improved, if he were to receive medical attention. Ben’s HS is greater than Anna’s. Yet, since Anna can recover fully if she receives treatment, and since there are limits to what can be done to treat schizophrenia, her CB is greater than Ben’s. How should one think about the health needs of Anna and Ben? If one were to adhere to aggregative approaches to relating health need factors to one another in order to generate determinate answers, whichever they may be, it must be determinately true that Anna is in greater, lesser, or equal need compared to Ben. This seems, however, not to be the case.

To see this, one might assume further that the best aggregation of the factors possible would entail that the health needs of Anna and Ben are of equal magnitude (if the example is not convincing, one might think of the instance that according to determinacy assumptions *must* exist where two persons with dissimilar health shortfalls and capacities to benefit have equal health needs). For the sake of argument, one can assume that Anna’s health need = {HS_A_ = 20; CB_A_ = 20}, and that Ben’s health need = {HS_B_ = 50; CB_B_ = 5}, and that the application of the best aggregation of the factors possible give: {HS_A_ = 20; CB_A_ = 20} = {HS_B_ = 50; CB_B_ = 5}. Assuming that all other factors relevant for establishing health needs are equal, using this concept of health need in a principle of need would lead to the conclusion that, prima facie, one ought to treat either Anna or Ben. Their needs are equal, and the right choice is made regardless of to whom the resources are allocated.

Now, enter Charles. As it happens, Charles was in the same accident as Anna and contracted exactly the same injury. However, he also has a bruise on his shoulder. So, his HS is *slightly* greater than Anna’s. Otherwise, Charles is perfectly healthy and has the same age and life expectancy as Anna. Charles’s health need = {HS_C_ = 20.00001; CB_C_ = 20}. It appears uncontroversial to say that Charles is in worse health than Anna and that he has a greater health need: {HS_C_ = 20.00001; CB_C_ = 20} > {HS_A_ = 20; CB_A_ = 20}. But is it really obvious that Charles has a greater health need than Ben? If one accepts the trichotomy thesis and embraces the idea that there must be an aggregation that puts the factors HS and CB together in a determinate way, then this must follow under the assumptions that have been made. Anna’s and Ben’s health needs are equal (since neither Anna’s nor Ben’s need is larger than the other’s, and the trichotomy thesis has been assumed), and so, if Charles’s health need is greater than Anna’s, it must also be greater than Ben’s. Yet, that seems to be false. A small change in HS such as a bruised shoulder cannot establish and determine whether a larger CB is more important than a larger HS when health needs are being compared.

Small changes in a single factor cannot change the comparative relation between alternatives when they meet that which speaks in their favor in fundamentally different ways. Anna and Ben do not have health needs of equal magnitude. The relation between their health needs is indeterminate in light of the trichotomy thesis. One plausible interpretation of this is that the relation is in fact parity. The relation between Anna’s and Ben’s health needs is symmetrical but irreflexive and non-transitive. This explains how Charles’s needs at the same time can be greater than Anna’s, but have the same relation to Ben’s needs as Anna’s needs have: parity is not a transitive relation. A different interpretation is that the concept of health needs is semantically vague so that it is, like with baldness, sometimes impossible to determine which need is the greater. Regardless of what the best explanation is, the trichotomy thesis should be rejected when the factors that a reasonable principle of need rely on are put together.

## Dealing with indeterminacy

What are we to do with principles that generate indeterminate evaluations? In particular, how should we treat the principle of need if it, as I have suggested, generates indeterminate evaluations? How to deal with indeterminacy is a topic that has received increasing attention in various research fields lately [26, 27]. In this section, I will discuss three different approaches to this issue with respect to a reasonable principle of need: (1) attach some other value to the principle of need that only applies to, is lexically inferior to, the principle of need; (2) impose determinacy; (3) empower decision makers and rely on their discretion for prioritization decisions. I will not defend any of these approaches, but rather, show how they *can* deal with the problem and point to some of their shortcomings. But before turning to the approaches, two points must be made.

First of all, it is important to see that indeterminacy by no means is a conclusive reason not to accept a principle [21]. Also a principle that generates indeterminate evaluations can be useful and pose constraints on rational choices. If the indeterminacy is only partial, there can be cases in which the application of the principle generates determinate answers. When the application of a principle generates indeterminate answers, those answers can be used to identify a set of not impermissible alternatives, and thereby exclude *some* courses of action from the set of permissible alternatives. In rational choice theory, one way of seeing such sets of permissible alternatives has been in terms of *Schwartz sets* [28]. Yet, indeterminate principles come with the problem of how to select between alternatives, the relation between which is indeterminate. Indeterminacy is not equality, so it is inadequate to conclude that it is rational to choose any of the alternatives, the relation between which is indeterminate.

Second, it is important to recognize that there actually are normative reasons to look for a systematic way of dealing with indeterminacy. In rational choice theory, it is often suggested that in the face of indeterminacy, one should simply accept that any element in the Schwartz set is permissible, and that nothing more can be said on that. In one sense, it is of course true that alternatives, the relation between which is indeterminate, are also permissible in the context of a principle of need. The principle of need cannot determine that it is impermissible to prioritize Anna over Ben. However, leaving matters at that comes with certain risks that are severe enough to warrant more systematic approaches to indeterminacy. The principle of need is supposed to be applied across a vast number of different cases. Accepting that the principle generates indeterminate conclusions and that one is free in such cases to choose among any of the alternatives means permitting discrimination. Such acquiescence would allow for certain decision patterns to arise, for certain conditions to be systematically prioritized in cases of indeterminacy, and for biases and prejudices to influence priority setting decisions. In order to avoid these risks, it is desirable to have a systematic approach to indeterminacy in priority setting situations and the application of the principle of need. Such a systematic approach might not reflect right-making features in light of the principle of need and its motivation (such right-making features that fully determine a ranking might not exist in case the principle is truly indeterminate), but it might be justified in light of norms that apply to decision processes.

One way to deal with indeterminacy problems is to introduce a decision-guiding value that can specify what to do in situations where the relation between some alternatives is indeterminate. For example, it could be suggested that in cases of indeterminacy, one ought to rely wholly on, for example, some welfarist principle in order to form a decision. In the case introduced above, this could mean that when one decides whom to prioritize, Anna or Ben, one ought to base this decision on how welfare can be maximized: is it by treating Anna or is it by treating Ben? Likewise, because the relation between the health needs of Ben and Charles is also indeterminate, one could base this decision of whom to prioritize on welfare maximization. One could introduce any normative principle that conclusively ranks the alternatives trichotomously to decide how to prioritize in situations of indeterminacy. It could here even be suggested that indeterminacy always should be dealt with by randomization so that every time the relation between two (or more) alternatives is indeterminate, they should be ranked trichotomously randomly, perhaps by rolling a dice.

Appendicizing the principle of need in this manner clearly deals with the theoretical decision problems that arise when different need factors must be considered together. However, the appeal of this type of solution is doubtful. First, if we are looking for a principle of need, we want a principle based on just that: *need*. Appendicizing a principle of need with values that have nothing to do with need is a big step away from the purpose of having the principle in the first place, and the question arises: is an appendicized principle of need really a principle of need at all? One’s view on this issue is likely to at least partly depend on whether one believes that severity-weighted need satisfaction matters non-derivatively or whether its importance is grounded in some other normative consideration or background theory. In case the principle of need itself is grounded in other normative considerations (perhaps it is defended with reference to how it leads to maximization of wellbeing), these normative considerations could with some plausibility be used to guide decision making in the face of indeterminacy. Yet, even if one accepts that an indeterminate principle of need maximizes wellbeing (or meets some other value that matters), it is not obvious that a principle of need appended with the value that it is grounded in meets that value in a way that is better compared with the alternative ways of dealing with indeterminacy, e.g., randomizing decision making, letting equality guide decisions. Whether it is better is an empirical question that cannot be answered at a theoretical level. In case one believes that severity-weighted need satisfaction matters non-derivatively, it is hard to see how appendicizing the principle of need with a value that is unrelated to need can be defended.

Second, regardless of whether one embraces the principle of need non-derivatively or not, introducing a value (V) that only guides one’s decisions in case of indeterminacy allows for the following very troubling possibility: if the relation between Anna and Ben is indeterminate, V should guide the decision. Anna might meet V better than Ben, so Anna should be selected over Ben. Enter Charles. Charles, as indicated above, has a greater health need than Anna and the relation between his and Ben’s health need is indeterminate. Now, Ben might meet V better than Charles, so Ben should be selected over Charles. Yet, Anna, who has a determinately lesser health need than Charles, meets V better than Ben. Here, there is a cyclical evaluation of the three alternatives, and introducing V is even less decision guiding than not doing so (Anna is here a permissible alternative). A cyclical evaluation like this could appear, for example, if V is equated with the total sum of wellbeing and Anna is a woman with a vast social network that would benefit if she were helped, Ben is a man with a large family that benefits by him being helped, and Charles is a loner. Clearly, cyclical evaluations of this sort are undesirable, and they might not be easily avoided by approaches that suggest the introduction of a lexically inferior value that is decision guiding in the face of indeterminacy.

Another approach to indeterminacy would be to *impose* determinacy on the indeterminacy on a theoretical level and defend a specific aggregation approach to the different factors. Clearly, one *can* put HS and CB together in a determinate way. It is not difficult to come up with aggregation methods that do this, which I also illustrated in the previous section. One could argue that determinate usability is so important, that the value of having a truly useable principle of need is so high, that it outweighs the fact that the principle itself is imperfect and flawed. This would be to bite the bullet and to accept that small changes really can change the comparative relation between dissimilar alternatives.

Imposing determinacy clearly provides a solution to the theoretical problem with indeterminacy. However, the price for this is significant. If the relation between certain needs really is indeterminate, then imposing determinacy will institutionalize a type of unfairness. Ben and Charles might not have an equal claim on health resources when the relation between their health needs is indeterminate, but it does not follow from that that it is acceptable to impose determinacy so that Ben’s needs are held to be smaller than Charles’s. If the relation between Ben’s and Charles’s health needs really is indeterminate, then Ben is treated unfairly if, for decision-technical reasons, his health needs are treated as lesser than those of Charles. The fact that the relation between A and B is indeterminate does not mean that a decision pattern can be justifiably imposed on a large number of instances where A and B stand against each other so as to always favor one over the other. In other words, imposing determinacy fails to take all the factors into account in an appropriate way.

Finally, one could accept the indeterminacy of the principle such as it is and leave it to decision makers to deal with it as they encounter decision problems. In the context of the parity of practical reasons, Ruth Chang has suggested that individuals can *create* reasons that break the indeterminacy by *committing* to certain values [26, 29]. The reasons to pursue a career as a lawyer and the reasons to pursue a career as an academic philosopher might be on par for an individual, and this could be dealt with by the individual committing to one of the two careers, thereby creating new reasons that render one alternative determinately better than the other. Similarly, one could suggest that decision makers can create reasons that can break the indeterminacy when the principle of need is applied by committing to certain values. A physician who must ration his time between Anna and Ben could commit to a certain value such as equality in order to reach a determinate evaluation of the alternatives; treating Ben would create a more equal outcome. This solution resembles appendicizing the principle of need, but instead of providing an appendix, one lets decision makers create decision grounds. Although this might sound radical, there seem to be some reasons to pursue this path. It could be argued that medical practitioners and other decision makers who work with priority setting on a daily basis are more apt to make these kinds of decision than ethicists and policy makers who generally do not really know what is going on, who generally do not have the same experience, and who generally lack the special type of wisdom and sensitivity that can come only from having practiced something.

Partially leaving prioritization decisions to decision makers’ discretion is, however, also problematic. Leaving the issue to decision makers entails accepting that one and the same principle can be correctly applied to identical situations and generate different results. On a theoretical level, this is problematic because it appears to violate the ideal of practicality; the application of one and the same principle can generate contradictory conclusions depending on who applies it. On a practical level, it is problematic because persons with health needs would be treated differently when they seek medical attention depending merely on who happens to work at the moment. This violates a deeply rooted and widespread idea of justice as equal treatment. Furthermore, even if some decision makers with vast experience might be very wise and well-suited to make these decisions, allowing for this also means allowing for not so wise, not so well-suited, decision makers to make decisions. Prejudices and biases are widespread in our societies, and the risk that such will affect how decision makers deal with indeterminacy is overwhelming. Finally, the fact that someone is skilled in assessing health shortfalls and capacity to benefit is not a guarantee that she is also skilled at making the normative evaluations required to deal with indeterminacy.

## Conclusion

I have argued that any reasonable principle of need designed to deal with priority setting must rely on a conception of health need that takes this to be a composite property composed of different factors. I suggested two factors that come together and form health need: health shortfall and capacity to benefit. When the relation between these factors is scrutinized, it proves to be partially indeterminate. This means that there will be situations in which it is false that one person has a smaller, larger, or equal health need compared with another person. The application of a reasonable principle of need will, thus, occasionally generate indeterminate conclusions. This poses serious challenges for proponents of principles of need as well as for theorists who try to include health need among the normative grounds for priority setting.

There are additional problems with principles of need that I have only touched upon in this article but that relate to the discussion above. Most obviously, these relate to the fact that a principle of need will not be the only principle guiding priority-setting decisions. There are good reasons to be a pluralist when it comes to normative standards for priority-setting decisions. Health needs matter, but so does, for example, equality. We must figure out how the principle of need relates to other standards, and that is no easy task. The argument I have presented for indeterminacy is generalizable, and it would not be surprising to find indeterminacy also when, for example, how health needs and equality relate to each other is scrutinized.

In conclusion, the theoretical problems surrounding how to put different health need factors together mainly show the following: any reasonable principle of need must be placed in a wider normative context. A principle of need cannot settle all priority-setting decisions because health need is not the only thing that matters when allocating scarce health resources. When the principle of need is scrutinized, another reason becomes apparent for why need is only one among many aspects that matter: no reasonable principle of need can be consistently useable unless there are other principles that can guide decision making when the application of the principle of need generates indeterminate conclusions.
